# Facilitating and Inhibiting Factors of Sexual Behavior among Migrants in Transition from Mexico to the United States

**DOI:** 10.3389/fpubh.2017.00149

**Published:** 2017-06-30

**Authors:** Jesús Alejandro Guerra-Ordoñez, Raquel A. Benavides-Torres, Rogelio Zapata-Garibay, Dora Julia Onofre-Rodríguez, María Aracely Márquez-Vega, Gabriela Zamora-Carmona

**Affiliations:** ^1^Universidad Autónoma de Tamaulipas, UAMM, Matamoros, México; ^2^Universidad Autónoma de Nuevo León, FAEN, CIDICS, Monterrey, México; ^3^United States—México Border Health Commission, Tijuana, México; ^4^Colegio de la Frontera Norte, Monterrey, México

**Keywords:** migrants, HIV, sexual behavior, transition, safe sex

## Abstract

**Introduction:**

Human immunodeficiency virus/acquired immune deficiency syndrome (HIV/AIDS) is one of the most prevalent infectious diseases in the border region of Mexico due to the flow of migrants under desperate conditions, encouraging casual and unprotected sex. Since this has become a binational public health problem, it is important to understand the factors that predict these sexual behaviors. The aim of the current study was to investigate the facilitators and inhibitors of transition in the sexual behavior of migrants from two border regions on the Mexico–United States (US) border.

**Methods:**

This was a predictive and cross-sectional study. A sample of 256 migrants in shelters for migrants on the border between Mexico and US were selected through systematic random sampling. Predictor variables investigated for effect on the safe sexual behavior (SSB) of the migrant were reasons for having sex; sexual attitudes; sexual machismo; knowledge about HIV; access to health services; and social discrimination.

**Results:**

The sample was predominantly male (89.5%), with 46.1% reporting being single. The average age was 33.38 years (SD = 9.73) and the average number of years of education reported was 8.05 (SD = 3.37). A permissive sexual attitude and sexual machismo both correlated with condom use (*r*_s_ = 0.130, *p* < 0.01 and *r*_s_ = −0.174, *p* < 0.01, respectively). Regression analysis showed that a permissive sexual attitude decreased the practice of safe sex (β = 0.17, *t* = 4.16, *p* < 0.001), as did sexual machismo (β = −0.28, *t* = −4.83, *p* < 0.001) and HIV knowledge (β = −0.11, *t* = −2.62, *p* = 0.006).

**Discussion:**

It was found that access to health services did not influence the SSB of migrants, as suggested by the literature. However, a permissive sexual attitude, sexual machismo, and HIV knowledge were all variables capable of predicting SSB. It is recommended that the study is extended to study migrant populations from other parts of the border, as well undertaking as a qualitative approach to explore new variables.

## Introduction

Human immunodeficiency virus/acquired immune deficiency syndrome (HIV/AIDS) is among the most prevalent infectious diseases in the border region of Mexico (1). Some authors have suggested that the HIV epidemic is centered in certain social groups, such as injecting drug users, female sex workers, and men who have sex with other men. The National Human Rights Commission (CNDH) reports that HIV is on the rise within some marginalized social groups, of which migrants are among the most at risk (2). According to El Colegio de la Frontera Norte (El COLEF), in 2015 more than 73,000 migrants traveling from the south with the intention of crossing into the United States (US) were detected. Several traveling groups of national and foreign migrants converge at the northern border of Mexico, which increases the number of these groups in the region (3).

Increasing migration flows in the country increase the vulnerability of those involved due to overcrowded conditions, which may itself compromise health, as well as favoring casual and unprotected intercourse (2). The conditions in which migrants strive to survive propitiate sexual behaviors at risk of acquiring HIV/AIDS infections (4). Such sexual behaviors have been associated with both personal and environmental factors, such as attitudes toward sexuality, and it has been reported that about half (40%) of the migrants agree to have sex with occasional sex partners, and more than 50% agree to have sex in exchange for money (5, 6).

Another influential factor found in the sexual behavior of migrants is cultural attitudes, such as machismo. Studies reveal that about 60% of married migrants who have sex with female sex workers (FSW) consider that, as men, they are entitled to satisfy their sexual needs while they are apart from their spouses (4, 6). In addition, associations have been shown between levels of machismo and sexual practices (7). Likewise, sexual attitudes are an important variable that define sexual behavior, with a previous study showing that more than 90% of participants have sexual attitudes that favor some types of sexual behavior (8).

Poor, or lack of, knowledge, about HIV is a relevant factor because it has been associated with increasing likelihood of engaging in risky sexual behavior. Indeed, around 50% of migrants believe that HIV can be transmitted by eating with the same utensils as another person or through mosquito bites (5, 6, 9). On the other hand, studies reveal that migrants with knowledge of HIV prevention present safer sexual behaviors (10–12).

In terms of environmental aspects, community and social factors are important. Limited access to health care and educational material on health protection is among the community factors that influences the sexual behaviors of migrants and has been associated with acquiring HIV/AIDS. Some studies demonstrate that lack of access to health care is linked to risky sexual practices, because of inconsistent condom use (13, 14). On the other hand, discrimination and social exclusion are important social factors. It has been reported that approximately 40% of migrants tend to stay in shared housing in precarious conditions and are not able to integrate adequately into the society in which they live (5).

Considering all this information, the factors described above may be considered to act as potential predictors of safe sexual behavior (SSB). Based on this, the construct of facilitators and inhibitors within a theory of transitions was devised as theoretical support (15). In this case, migration is considered a transition that is affected by the following personal variables (1) reasons for having sex, including feeling good, intimacy, and affirmation; (2) sexual attitudes, including permissiveness, communion, and instrumentality; (3) sexual machismo; and (4) HIV knowledge. Environmental variables include (1) access to health services and (2) social discrimination. These factors are considered to influence SSB either as facilitators or inhibitors and to act as an indicator of SSB.

By incorporating variables predictive of the SSB of migrants, this study provides important insights into the problem of HIV prevalence in the northern border of Mexico; a phenomenon that is considered a binational problem of public health. Therefore, understanding the associations of SSB in this population is fundamental to reduce the risk of HIV and other sexually transmitted diseases (STDs) in Mexico and in the US. This work allows a series of research questions to be established, such as “Is there an association between reasons for having sex, sexual attitudes, or cultural beliefs (such as sexual machismo) and SSB in migrants?” “Does knowledge about HIV influence immigrants’ SSB?” and “Are environmental factors – such as discrimination or access to health services—associated with migrant workers’ SSB?” Based on these questions, the objective of this research was to test the effect of transition facilitators and inhibitors on SSB.

## Materials and Methods

### Design

This was a predictive and cross-sectional study conducted in migrant shelters on the US/Mexico border.

### Sampling and Recruitment

The sample was made up of migrants in transit to the US, as well as migrants deported to Mexico, from the cities of Reynosa and Matamoros (both border cities within the state of Texas). The total sample was 256 migrants housed in migrant shelters. The sample was based on a linear regression model of 10 variables with a coefficient of determination of 0.09, a test power of 95%, and a statistical significance of 0.05.

The migrants were selected according to a systematic random sampling of 1 in 4. Registration lists used for migrants when entering the shelter for migrants were accessed. The migrants had previously been assigned a consecutive control number. The first migrant to be included was the second number on the list, and then, every fourth migrant was approached to verify that he/she met the inclusion criteria. If the selected migrant did not meet these criteria, the count was restarted. If the migrant met the inclusion criteria, he/she was invited to participate in the project. If he/she refused, he/she was thanked and the procedure would continue. Consenting participants were instructed to move to the assigned area where the instruments were applied. Within this area, it was verified that the migrant was sexually active, with non-sexually active individuals being excluded from the study. The research process and objectives were explained to included migrants, as well as the risks and benefits of the study and the anonymous and voluntary nature of participation. In addition, participants were informed that all information would be handled confidentially, and that they would be identified by a folio number only, to which only researchers would have access.

### Ethical Considerations

This study was approved by the Ethics and Research Commissions of the School of Nursing (FAEN) of the *Universidad Autonoma de Nuevo Leon*, as well as the leaders of each of the migrant shelters. Respect for the dignity and protection of the rights and welfare of study subjects who agreed to participate in the research project were considered at all times. Protection of the subjects and their information was guaranteed before, during, and after the completion of the instruments, and all participants were housed in dignified and private facilities.

All participants were provided with an informed consent form, with which they gave their authorization to participate, in full knowledge of the nature of the procedures and risks to which they would submit. The consent was reviewed and approved by the Ethics Committee of the FAEN and contained the names of the researchers and the project, as well as two witnesses who endorsed the migrant’s participation. It also contained the purpose, procedures, risks and benefits, a guarantee of answers to questions, freedom to withdraw without fear of reprisals, security of confidentiality and the anonymity of all participants, and the guarantee to suspend the research immediately if the health of the migrants was at risk.

In addition, participants were given a clear and complete explanation of the study, so they could understand the important aspects of the research. This was carried out by health-care professionals with the knowledge and experience to ensure the integrity of the migrant. All collaborators who participated in the collection of data had previously successfully completed the National Institutes of Health online course, “Protection of Human Participants in Research,” on ethical aspects of research.

### Measurements

#### Sociodemographic Data Card

This document was structured by the researchers and contained sociodemographic variables, such as gender (male/female), age (years), education (grade of education completed), marital status (single/married/divorced/widowed/consensual union), and weekly income (in Mexican pesos).

#### Reasons for Having Sex

The Reasons for Having Sex scale (16), consisting of 29 items that contain various reasons for having sex, was used. The instrument has six subscales: “feel good,” “affirmation,” “intimacy,” “coping,” “approval of the couple,” and “approval of pair,” with a sample item being “satisfying sexual needs.” Response options ranged from 1 = almost never/never to 5 = almost always/always. The total score for each of the subscales ranged from 5 to 25. Higher scores indicated greater reason for having sex. From this instrument, we included the three following sub-scales: feel good, intimacy, and affirmation, which have reported Cronbach’s alpha of 0.87, 0.90, and 0.87, respectively.

#### Sexual Attitudes

We used a variation of the Brief Sexual Attitudes scale, translated and adapted for the cultural context (17). This scale has 23 items divided into four subscales (“permissiveness,” “communion,” “instrumentality,” and “birth control”), of which only the first three were used in this study. The instrument includes 20 items with a 5-point Likert scale response, ranging from 1 = strongly agree to 5 = strongly disagree. An example question would be, “It’s okay to have casual/occasional sex.” The results were analyzed according to average response, in which a low average represents greater sexual attitudes. The subscales have reported Cronbach’s alpha of 0.93, 0.71, and 0.77 for permissiveness, communion, and instrumentality, respectively.

#### Sexual Machismo

The Sexual Machismo scale was used for this measurement (18). This scale measures sexism in people and consists of 12 items which have a score ranging from 1 to 5. An example statement would be, “Only the man has a sexual experience,” with response options ranging from 1 = totally disagree to 5 = totally agree. The minimum score is 12 and the maximum is 60, with higher scores indicating greater sexual machismo. The scale has shown good internal consistency, with Cronbach’s alpha = 0.91.

#### HIV Knowledge

The HIV Knowledge Questionnaire (HIV-KQ-18) was used for this measurement (19). This questionnaire is used to measure knowledge about HIV transmission and prevention. The instrument consists of 18 items with “True,” “False,” and “I don’t know” answers. An example of a statement would be “There is a vaccine that prevents the adult person from becoming infected with HIV.” The instrument has a reported internal consistency of 0.75–0.89 and is considered to be stable and suitable for use with low-literacy populations.

#### Access to Health Services

The Accessibility to Health Services subscale of the Quality of Services of Secondary Health Care Centers scale (20) was used for this measurement. This scale is used to measure the quality of the health care services and comprises 29 items grouped in dimensions of reliability, empathy, responsiveness, accessibility, and tangibles. For this study, only one subscale, “accessibility” was used, consisting of five items with a 7-point Likert scale response ranging from “strongly disagree” to “strongly agree.” A sample statement would be, “Whenever I need it, I have access to medical tests that need to be performed.” The subscale has a Cronbach’s alpha of 0.80.

#### Social Discrimination

The Experienced Social Discrimination scale was used (21), which has eight items with a Likert scale response ranging from 1 = always to 5 = never. A sample question would be, “You are treated with less respect than others.” The minimum scale score is 8 and represents those who have experienced the situations of discrimination expressed in the items. The maximum score is 40 and represents those who have never experienced any of these discrimination situations. The internal consistency of the scale has been demonstrated by a Cronbach’s alpha of 0.81.

#### Safe Sex

Two sub-scales (condom use and safe sex) were translated and adapted from the Safe Sex Behavior Questionnaire (22). Each subscale consists of eight items in which participants are asked to respond according to how often they engage in sexual behavior; the surveys are on a Likert scale with the following options: 1 = Never, 2 = Sometimes, 3 = Most of the time, and 4 = Always. A sample statement would be, “I have anal sex without using a condom.” The instrument has a reported Cronbach’s alpha of 0.91.

### Data Analysis

Responses to instruments were captured electronically using the Qualtrics application for the operating system Android, which avoids omissions in the responses. The device loaded with the application stored the surveys and uploaded them to the online Qualtrics platform. During this period, no one could access the answers, except for the main researcher who had access to the online account secured with a password, to ensure that the answers were completely secure. Once the surveys were loaded on the platform, they were downloaded on to a computer in a format compatible with Statistical Package for Social Sciences (SPSS) for further analysis.

After obtaining the data in SPSS version 20.0, both descriptive and inferential statistics were used. Frequencies and percentages were used for some of the sociodemographic variables; for others, central tendency and dispersion measures were used, as well as normality Kolmogorov–Smirnov tests with a Lilliefors correction. Given the abnormal distribution of the results, we performed a correlation analysis with the Spearman coefficient. Later, in order to meet the objective of this work, which was to test the effect of the independent variables on dependent variables, we performed a discriminant analysis with Wilk’s lambda statistic to verify that the variables were not significant in the model. Once these were eliminated, a regression analysis was performed using a linear regression analysis with the variables that were significant in the model using a multiple generalized linear multivariate model with Bootstrap sampling performed.

## Results

Table 1 shows that the predominant gender was male (89.5%). In terms of marital status, 46.1% reported being single, 22.7% were married, 8.2% divorced, 1.2% widowed, and 21.9% had a consensual union. These results indicate that male and single migrants represent the largest migratory flow in this border region.

**Table 1:** Frequencies and percentages of sociodemographic data (*n* = 256).

	Frequencies	Percentage
**Sex**		
Female	27	10.5
Male	229	89.5
**Marital status**		
Single	118	46.1
Married	58	22.7
Divorced	21	8.2
Widowed	3	1.2
Consensual union	56	21.9

As shown in Table 2, the average age was 33.38 (SD = 9.73) years, with a minimum age of 18 and a maximum age of 58 years. The average number of years of education was 8.05 (SD = 3.37), with a minimum of 0 and a maximum of 15 years of study. Average income was 243.01 (SD = 754.97) Mexican pesos per week, with a minimum of 0 and a maximum of 5,600 Mexican pesos per week.

**Table 2:** Measures of central tendency and dispersion of sociodemographic variables.

Variable	M	Mdn	Mode	SD	Min	Max	*D*^a^	*p*-Value
Age	33.38	32.50	25.00	9.73	18.00	58.00	0.088	0.001
Education	8.05	9.00	9.00	3.37	0.00	15.00	0.119	0.001
Income	234.01	0.00	0.00	754.97	0.00	5,600.00	0.434	0.001

*D^a^ = Normality Kolmogorov–Smirnov test correction Lilliefors*.

Table 3 shows the central tendency measures of the independent and dependent variables and reveals that, of the reasons for having sex, “feel good” had an average score of 5.44 (SD = 31.31), “intimacy” had a score of 71.13 (SD = 31.85), and “affirmation” had a score of 48.18 (SD = 34.42). In terms of sexual attitudes, “permissiveness” scored 62.34 (SD = 23.99), “communion” scored 25.21 (SD = 22.78), “instrumentality” scored 50.27 (SD = 28.49), “sexual machismo” scored 24.96 (SD = 17.30), “HIV knowledge” scored 54.90 (SD = 19.83), “access to health services” scored 30.55 (SD = 35.37), and “social discrimination” scored 76.01 (SD = 23.07). Regarding the variables related to SSB, “condom use” scored 64.58 (SD = 21.45) and “safe sex” scored 78.27 (SD = 15.10). All variables showed a statistical significance of *p* < 0.05 and are, thus, considered without normal distribution.

**Table 3:** Measures of central tendency and dispersion of independent and dependent variables.

Variable	M	SD	Min	Max	*D*^a^	*p*-Value
Reasons for having sex						
Feel good	51.44	31.31	0.00	100	0.081	0.001
Intimacy	71.13	31.85	0.00	100	0.189	0.001
Affirmation	48.18	34.42	0.00	100	0.122	0.001
Sexual attitudes						
Permissiveness	62.34	23.99	10	100	0.074	0.001
Communion	25.21	22.78	0.00	100	0.153	0.001
Instrumentality	50.27	28.49	0.00	100	0.070	0.001
Sexual machismo	24.96	17.30	0.00	89.58	0.075	0.001
HIV knowledge	54.90	19.83	0.00	94.44	0.103	0.001
Access to health services	30.55	35.37	0.00	100	0.259	0.001
Social discrimination	76.01	23.07	0.00	100	0.149	0.001
Safe sexual behavior						
Condom use	64.58	21.45	12.50	100	0.109	0.001
Safe sex	78.27	15.10	33.33	100	0.093	0.001

*D^a^ = Normality Kolmogorov–Smirnov test correction Lilliefors*.

The Spearman coefficient was used for the correlation analysis. Table 4 shows correlations between the facilitators/inhibitors and SSB. It may be seen that the subscale “condom use” is related only to the sexual attitude “permissiveness” (*r*_s_ = 0.130, *p* < 0.01) and sexual machismo (*r*_s_ = −0.174, *p* < 0.01). In contrast, the “safe sex” subscale correlated with multiple independent variables, including reason for having sex: “feel good” (*r*_s_ = −0.209, *p* < 0.01); sexual attitudes: “permissiveness” (*r* = 0.434, *p* < 0.01), “communion” (*r*_s_ = 0.168, *p* < 0.01), and “instrumentality” (*r*_s_ = 0.280, *p* < 0.01); “sexual machismo” (*r*_s_ = −0.461, *p* < 0.01); and “social discrimination” (*r*_s_ = 0.204, *p* < 0.01).

**Table 4:** Correlation between facilitating and Inhibiting factors and safe sexual behavior.

	SSB	
Facilitators/inhibitors	Condom use	Safe sex
Reasons for having sex		
Feel good	0.067	−0.209**
Intimacy	0.085	0.084
Affirmation	0.083	−0.103
Sexual attitudes		
Permissiveness	0.130**	0.434**
Communion	0.063	0.168**
Instrumentality	−0.006	0.280**
Sexual machismo	−0.174**	−0.461**
HIV knowledge	0.119	−0.061
Access to health services	−0.030	0.040
Social discrimination	0.037	−0.204**

**p < 0.01.

A generalized linear model with facilitators and inhibitors as covariates and the sub-scales of SSB as dependent variables was used for the regression analysis. Table 5 shows a discriminant analysis with Wilk’s lambda statistic in the initial model, which includes all covariates and their statistical significance within the model. This model shows that the reasons for having sex do not reach statistical significance within the model for “feel good” (Λ = 0.99, *p* = 0.229), “intimacy” (Λ = 0.99, *p* = 0.424), or “affirmation” (Λ = 0.98, *p* = 0.117). Sexual attitudes “communion” (Λ = 0.98, *p* = 0.154) and “instrumentality” (Λ = 0.98, *p* = 0.112), as well as facilitators and environmental inhibitors, such as “access to health services” (Λ = 0.99, *p* = 0.821) and “social discrimination” (Λ = 0.98, *p* = 0.097) also do not reach statistical significance.

**Table 5:** Initial model’s discriminant analysis with Wilks’ lambda.

Effect	Λ	*F*	df of the hypothesis	df of error	*p*-Value
Intersection	0.59	84.22	2	244	0.001
Feel good	0.99	1.48	2	244	0.229
Intimacy	0.99	0.86	2	244	0.424
Affirmation	0.98	2.17	2	244	0.117
Permissiveness	0.96	5.46	2	244	0.005
Communion	0.98	1.88	2	244	0.154
Instrumentality	0.98	2.21	2	244	0.112
Sexual machismo	0.92	10.49	2	244	0.001
HIV knowledge	0.96	4.61	2	244	0.011
Access to health services	0.99	0.19	2	244	0.821
Social discrimination	0.98	2.36	2	244	0.097

*Λ = Wilks’ lambda*.

From these results, we can see that the covariates mentioned without statistical significance (*p* > 0.05) do not represent an effect on the SSB of the migrants. On the other hand, the sexual attitude “permissiveness” (Λ = 0.96, *p* = 0.005), sexual machismo (Λ = 0.92, *p* < 0.001), and HIV knowledge (Λ = 0.96, *p* = 0.011), all demonstrate statistical significance within the model and, therefore, can be shown to have an effect on SSB.

Table 6 shows the intersubject effects test and reveals that the corrected models are statistically significant; however, in individual terms, only the “permissiveness” sexual attitude has a statistically significant relationship with safe sex (*F*_[1,245]_ = 10.73, *p* < 0.001). Sexual machismo is statistically related to both dependent variables, “condom use” (*F*_[1,245]_ = 6.44, *p* = 0.012) and “safe sex” (*F*_[1,245]_ = 17.88, *p* < 0.001), while “HIV knowledge” shows a significant relationship with “safe sex” (*F*_[1,245]_ = 4.96, *p* = 0.027).

**Table 6:** Test of inter-subjects effects of the initial model.

	Dependent variable	Type III sum of squares	df	Mean square	*F*	*p*-Value
Corrected template	Condom use	10,581.16	10	1,058.12	2.43	0.009
	Safe sex	18,042.94	10	1,804.29	11.02	0.001

Intersection	Condom use	14,551.84	1	14,551.84	33.40	0.001
	Safe sex	25,645.51	1	25,645.51	156.68	0.001

Feel good	Condom use	33.16	1	33.16	0.08	0.783
	Safe sex	430.85	1	430.85	2.63	0.106

Intimacy	Condom use	491.47	1	491.47	1.13	0.289
	Safe sex	52.66	1	52.66	0.32	0.571

Affirmation	Condom use	752.61	1	752.61	1.73	0.190
	Safe sex	551.68	1	551.68	3.37	0.068

Permissiveness	Condom use	506.32	1	506.32	1.16	0.282
	Safe sex	1,756.03	1	1,756.03	10.73	0.001

Communion	Condom use	1,564.66	1	1,564.66	3.59	0.059
	Safe sex	99.76	1	99.76	0.61	0.436

Instrumentality	Condom use	901.25	1	901.25	2.07	0.152
	Safe sex	255.55	1	255.55	1.56	0.213

Sexual machismo	Condom use	2,808.10	1	2,808.10	6.44	0.012
	Safe sex	2,925.96	1	2,925.96	17.88	0.001

HIV knowledge	Condom use	1,151.98	1	1,151.98	2.64	0.105
	Safe sex	812.05	1	812.05	4.96	0.027

Access to health services	Condom use	128.30	1	128.30	0.29	0.588
	Safe sex	7.55	1	7.55	0.05	0.830

Social discrimination	Condom use	13.96	1	13.96	0.03	0.858
	Safe sex	720.81	1	720.81	4.40	0.037

Error	Condom use	106,745.23	245	435.69		
	Safe sex	40,102.61	245	163.68		

Total	Condom use	1,185,104.17	256			
	Safe sex	1,626,510.42	256			

Total corrected	Condom use	117,326.39	255			
	Safe sex	58,145.55	255			

*Sampling Bootstrap with 2,000 samples*.

In Table 7, we observe the final model after applying the discriminant analysis with the Wilk’s lambda statistic. Observing only the facilitators and inhibitors that present statistical significance within the model, it is shown that the sexual attitude “permissiveness” (Λ = 0.94, *p* < 0.001), sexual machismo (Λ = 0.91, *p* < 0.001), and HIV knowledge (Λ = 0.96, *p* = 0.07) are significant. An improved statistical significance of the co-variables was observed after the discriminant analysis, which indicates a greater effect on SSB.

**Table 7:** Final model’s discriminant analysis with Wilks’ lambda.

Effect	Λ	*F*	df of the hypothesis	df of error	*p*-Value
Intersection	0.42	176.27	2	251	0.001
Permissiveness	0.94	8.63	2	251	0.001
Sexual machismo	0.91	12.27	2	251	0.001
HIV knowledge	0.96	5.03	2	251	0.007

*Λ = Wilks’ lambda*.

Table 8 shows the intersubject effects test of the final model, from which it can be seen that the corrected models are statistically significant as in the initial model. Individually, the sexual attitude “permissiveness” is statistically significantly related to “safe sex” (*F*_[1,252]_ = 17.30, *p* < 0.001), sexual machismo with both dependent variables, “condom use” (*F*_[1,252]_ = 3.93, *p* = 0.049), and “safe sex” (*F*_[1,252]_ = 23.30, *p* < 0.001), and HIV knowledge is significantly related with “safe sex” (*F*_[1,252]_ = 6.86, *p* = 0.009). As in the previous model, it is observed that for safe sex, the three co-variables (sexual attitude “permissiveness,” sexual machismo, and knowledge about HIV) have an effect; nevertheless, for the use of the condom only, the sexual machismo has an effect in this case.

**Table 8:** Test of inter-subjects effects of the final model.

Origin	Dependent variable	Type III sum of squares	df	Mean square	*F*	*p*-Value
Corrected template	Condom use	5,690.29	3	1,896.76	4.28	0.006
	Safe sex	16,063.90	3	5,354.63	32.06	0.001

Intersection	Condom use	30,907.33	1	30,907.33	69.77	0.001
	Safe sex	54,455.82	1	54,455.82	326.10	0.001

Permissiveness	Condom use	354.55	1	354.55	0.80	0.372
	Safe sex	2,889.35	1	2,889.35	17.30	0.001

Sexual machismo	Condom use	1,741.26	1	1,741.26	3.93	0.049
	Safe sex	3,891.18	1	3,891.18	23.30	0.001

HIV Knowledge	Condom use	768.01	1	768.01	1.73	0.189
	Safe sex	1,145.08	1	1,145.08	6.86	0.009

Error	Condom use	111,636.10	252	443.00		
	Safe sex	42,081.65	252	166.99		

Total	Condom use	1,185,104.17	256			
	Safe sex	1,626,510.42	256			

Total corrected	Condom use	117,326.39	255			
	Safe sex	58,145.55	255			

*Sampling Bootstrap with 2,000 samples*.

The model that has as a dependent variable the use of the condom explains a variance of 3.7%, while the model with the dependent variable as safe sex accounts for 26.8% of the variance.

Table 9 shows that migrants with poorer safe sex practices have a permissive sexual attitude that is 0.17 less than migrants who do not have this sexual attitude (β = 0.17, *t* = 4.16, *p* < 0.001), a sexual machismo that is 0.28 less than those without sexual machismo (β = − 0.28, *t* = −4.83, *p* < 0.001), and a knowledge about HIV that is 0.11 less than those with greater knowledge about HIV (β = −0.11, *t* = −2.62, *p* = 0.006).

**Table 9:** Parameter estimations.

Dependent variable	Parameter	*B*	Std. error	*p-*Value	CI 95%	
					Lower	Upper
Condom use	Intersection	60.63	7.47	0.001	45.89	75.32
	Permissiveness	0.06	0.07	0.418	−0.09	0.21
	Sexual machismo	−0.18	0.09	0.058	−0.38	0.01
	HIV knowledge	0.09	0.07	0.180	−0.04	0.22

Safe sex	Intersection	80.47	4.67	0.001	71.69	89.46
	Permissiveness	0.17	0.04	0.001	0.08	0.26
	Sexual machismo	−0.28	0.06	0.001	−0.40	−0.17
	HIV knowledge	−0.11	0.04	0.006	−0.18	−0.03

*IC, confidence interval*.

## Discussion

Safe sexual behavior is a variable that can be influenced by multiple factors, both personal and environmental, all of which may encourage its presence or inhibit it. Migration involves multiple factors that are linked to an individual’s decision to participate in behaviors and activities that increase the risks of HIV infection (23). Zhuang et al. (6) found that migrants engaged in unsafe sexual behaviors, such as sex with FSW, for reasons such as physical and emotional sensations. This was similar to our study, given that the reason for having sex of “feeling good” had a correlation with safe sex. However, it should be noted that in the regression analysis, this had no effect on sexual behavior.

In a study by Li et al. (8), it was found that sexual attitudes in young migrants were associated with more frequent sexual practices; similar to our study, since the sexual attitudes of communion and instrumentality are related to safe sex. However, they do not represent predictive variables for SSB, as opposed to the sexual attitude of permissiveness, which had a correlation with both SSB variables and also a predictive effect on it. It should be mentioned that Li et al.’s study does not report whether the sexual relations practiced by young migrants were considered safe or risky.

A study published by Knipper et al. (7) performed in Latin migrants found an association between traditional masculine norms, such as machismo with the use of the condom, which was similar in this study since sexual machismo had a correlation with use of the condom as well as with safe sex, which itself proved to be a predictive variable for SSB. Similarly, a study by Organista et al. [cited in Magis-Rodríguez et al. (24)] indicated that Latino migrants show low initiative and a lack of capacity to negotiate condom use during sexual intercourse.

Furthermore, the qualitative study by McQuiston and Gordon showed that in newly immigrant Mexicans, condom use was also associated with trust in the couple, gender roles, perceptions of safe sex, and time. It is important to consider these aspects in interventions for HIV and STD prevention (25).

With regard to the HIV knowledge variable, a study by Knipper et al. (7) reported that greater knowledge about HIV transmission and prevention was found with condom use. Similar results were reported by Weine and colleagues (10), who reported that correlation between HIV protection and condom use was found in migrants who were most knowledgeable about HIV. Unlike these studies, we found no correlation between HIV knowledge and condom use. Similarly, Triuneh and colleagues (11) reported that surveyed migrants who had received information about HIV in the past 6 months were more likely to use a condom during their last non-marital sexual experience than those who had not. A study by Rhodes and colleagues, centered on the effectiveness of an HIV prevention intervention to increase condom use and HIV testing among heterosexual Latino immigrants, found that HIV participants engaged in prevention intervention reported a higher probability of consistent condom use and HIV testing (26).

On the other hand, another study (12) found an association between knowledge about AIDS and high sexual risk. Although there was a correlation between HIV knowledge in this study and some SSB, it represented a predictor variable for safe sex within the regression analysis, as mentioned in the latter study.

Glasman et al. (13) conducted an investigation into the disadvantages of accessing services for HIV prevention, where they found inconsistency in condom use in those subjects who did not have access to HIV preventive information. Meanwhile, another study (14) found that less than half of the subjects interviewed were affiliated with a sexual health and reproduction program and that a low proportion used condoms. Despite this evidence, access to health services of any type had no association with any SSB and was not a predictor of SSB in this study.

Finally, a qualitative study (27) evaluated the sexual behavior of migrants with respect to stigma and discrimination toward people with HIV. It was linked to poor knowledge about HIV, with people not understanding exactly how it was transmitted. It should be mentioned that this type of discrimination differs with respect to the measure in this investigation since it alludes to social discrimination in general. However, it was found that there was a link between general discrimination and safe sex, although it was not presented as a predictive variable within the analysis of regression.

It is recommended that this study be extended to migrant populations from other border regions of Mexico and the US to verify that the predictor variables are the same as those found in this study, or at least identify differences. A qualitative approach in this same population group may help to consider variables that are not included in this study, as well as research into the prevalence of sexually transmitted infections. These results allow us to consider how personal and environmental factors can influence the migrants’ SSB, which is fundamental to reduce HIV/STD risk in migratory contexts. In this sense, this study makes a contribution to health promotion by helping us to understand what factors are facilitators and inhibitors of SSB that may favor or limit the well-being and maintenance of the sexual health of migrants.

### Limitations

One limitation of this study is that few environmental variables were considered within the model, meaning that after the discriminatory analysis, none remained as predictors. In addition, the cross-sectional design restricts continuity in the case of any intervention. Finally, some of the questions were uncomfortable for the participants, so there is the potential for them to refuse to respond to the surveys.

### Transcendence

The present study allows us to know a little about the dynamics of the sexual behaviors of migrants, suggesting that multiple previously unknown factors have a considerable influence in this regard. This approach is useful to develop plans and develop culturally appropriate strategies for this specific population with regards to sexual health. In the future, efforts will be made to obtain funding for HIV/STD opportune detection, as well as to facilitate protective methods for safer sexual practices that will help improve health indicators in the region, especially considering that it is a vulnerable population and likely to increase with political and economic changes in both countries.

## Ethics Statement

This study was carried out in accordance with the recommendations of Ethics and Research Commissions of the School of Nursing (FAEN) of the Universidad Autónoma de Nuevo Leon with written informed consent from all subjects. All subjects gave written informed consent in accordance with the Declaration of Helsinki. The protocol was approved by the Ethics and Research Commissions of the School of Nursing (FAEN) of the Universidad Autónoma de Nuevo Leon.

## Author Contributions

JG-O is the intellectual author of the research idea, participated in the data analysis, and acted as primordial redactor of the manuscript. RB-T participated in much of the writing and design of the study. RZ-G participated in the writing of the statistical analysis. DO-R edited the discussion and design of the study. MM-V edited the conclusion. GZ-C participated by giving ideas for the design, suggesting methodology, and providing framework observations.

## Conflict of Interest Statement

The authors declare that the research was conducted in the absence of any commercial or financial relationships that could be construed as a potential conflict of interest.
